# Sociodemographic Profile of Pediatric Hemodialysis during the COVID-19 Pandemic and Challenges for Sustainable Development

**DOI:** 10.1590/0034-7167-2024-0041

**Published:** 2025-10-27

**Authors:** Nathália Cardoso Neves, Richarlisson Borges de Morais, Isis Oliveira Arruda, Maria Luiza Dautro Moreira do Val, Ana Lúcia Cardoso Santos Abreu, Maria Cristina de Andrade, Paulo Henrique Braz-Silva, Monica Taminato

**Affiliations:** I Universidade Federal de São Paulo. São Paulo, São Paulo, Brazil; II Universidade Federal de Uberlândia. Uberlândia, Minas Gerais, Brazil; III Universidade Federal de São Paulo, Hospital São Paulo. São Paulo, São Paulo, Brazil; IV Universidade de São Paulo. São Paulo, São Paulo, Brazil

**Keywords:** Pediatrics, Hemodialysis, COVID-19, Demographic Factors, Sustainable Development Goals, Pediatría, Hemodiálisis, COVID-19, Factores Demográficos, Objetivos de Desarrollo Sostenible

## Abstract

**Objectives:**

to relate sociodemographic characteristics with SARS-CoV-2 serology of minors under hemodialysis and their caregivers.

**Methods:**

a cross-sectional, descriptive study with anti-SARS-CoV-2 Spike immunoassay analysis.

**Results:**

ten patients and their companions were included before vaccination against COVID-19. The overall seroprevalence was 25% and 20% among patients, who had a mean age of 11.47 years, 70% of whom were male. All companions were women (100%), with a mean age of 38.41 years and 60% of mixed race. Half (50%) receive between 1 and 3 minimum wages, and 70% spend more than two hours commuting. As for the residences, all (100%) are made of masonry and have basic sanitation.

**Conclusions:**

the importance of routine tracking of COVID-19 cases and the impact of socioeconomic variables on the quality of life of children and family members is evidenced.

## RESUMEN

**Objetivos:**

relacionar las características sociodemográficas con la serología del SARS-CoV-2 de menores en hemodiálisis y sus cuidadores.

**Métodos:**

estudio descriptivo transversal con análisis de inmunoensayo anti-SARS-CoV-2 Spike.

**Resultados:**

se incluyeron diez pacientes y sus acompañantes, antes de la vacunación contra la COVID-19. La seroprevalencia global fue del 25% y del 20% entre los pacientes, que tenían una edad media de 11,47 años, y el 70% eran varones. Todos los acompañantes eran mujeres (100%), con una edad media de 38,41 años y un 60% de raza mestiza. La mitad (50%) recibe entre 1 y 3 salarios mínimos y el 70% pasa más de dos horas desplazándose. En cuanto a la residencia, todas (100%) son de mampostería y cuentan con saneamiento básico.

**Conclusiones:**

se evidencia la importancia del seguimiento rutinario de los casos de COVID-19 y el impacto de las variables socioeconómicas en la calidad de vida de los niños y las familias.

## INTRODUCTION

Chronic kidney disease is a public health concern with a significant increase in incidence and prevalence worldwide, and is associated with increased morbidity and mortality. It is characterized by the gradual, progressive and irreversible loss of kidney function, especially glomerular filtration capacity. The progression of the condition leads to kidney failure and the need for Renal Replacement Therapy (RRT), with dialysis (hemodialysis (HD) and peritoneal dialysis (PD)) and kidney transplantation currently available(1,2).

Although RRT methods offer a significant increase in survival, they can also present metabolic, cardiovascular and infectious complications, in addition to compromising the growth, development and quality of life of children and their families. An epidemiological study that gathered global data indicates that children undergoing HD have mortality rates 30 times higher than healthy children, with the main causes being cardiovascular diseases (30% to 40%) and infections (20% to 50%)(3). Another study reveals that these mortality rates can increase 30 to 150 times and that life expectancy is approximately 50 years less than the pediatric population without comorbidities(2).

With the advent of the COVID-19 pandemic emergency, declared in March 2020 by the World Health Organization, the challenges faced by children and adolescents undergoing HD have become even more complex. This new disease is caused by the SARS-CoV-2 virus, of the *Coronaviridae* family, transmitted by aerosols and contaminated objects, causing Severe Acute Respiratory Syndrome by SARS-CoV-2(4,5,6,7).

Literature reviews indicate that children have a good prognosis for the disease, with recovery within two weeks and mild to moderate symptoms, with the most frequent symptoms being fever, dry cough, fatigue, nasal congestion and runny nose. Moreover, they indicate that most transmissions occurred through close contact with family members or people who tested positive for COVID-19(4,8,9,10). A pediatric study that included data from China, Italy, and the United States showed that children represent a variation of 1% to 5% of confirmed cases of COVID-19, with 2% of cases in China (4.672), 1.2% in Italy (22.500), and 5% in the United States (4.226). Of this amount, only two cases of death were identified in children diagnosed with COVID-19(8).

Concerning children with chronic kidney disease, an Egyptian study found an incidence rate of 16.2% of COVID-19 among those undergoing HD and a mortality rate of 15.7% in those undergoing RRT. The study attributes this incidence to the fact that this population is unable to comply with one of the main measures to prevent exposure to the SARS-CoV-2 virus, social distancing, given the need to travel to HD sessions multiple times a week, in addition to routine health checkups and exams(11).

The 17 Sustainable Development Goals (SDGs) are global targets set by the United Nations (UN) by 2030 to eradicate poverty, protect the planet and ensure peace and prosperity for all people(12). Access to quality healthcare services, such as HD, plays a crucial role in achieving SDG 3 – Good Health and Well-being.

Furthermore, support and education provided to the families of these children plays a critical role in helping to mitigate emotional and financial stress associated with long-term treatment. Ensuring equitable access to quality healthcare services for all children, regardless of their medical condition or geographic location, is essential to promoting not only their health but also the sustainability of communities and societies as a whole(13).

Considering the SDGs established by the UN, it is clear that children undergoing HD constitute a vulnerable group, not only due to health issues, but also to sociodemographic and economic challenges related to daily life. Therefore, their companion/family caregiver was also included in screening, considering that the outpatient HD environment is shared and that there is great proximity and coexistence between these agents in the care of children in this population. From this perspective, it is essential to investigate the occurrence of COVID-19 and the sociodemographic variables that impact quality of life and access to healthcare of this group. In this way, scientific knowledge can support strategies and actions that lead to the goals of Eradicating Poverty (SDG 1), Good Health (SDG 3) and Reducing Inequalities (SDG 10), providing a dignified existence for this population(13).

## OBJECTIVES

### General objective

To analyze the sociodemographic characteristics of pediatric HD patients and their family caregivers and relate them to SARSCoV-2 serology.

### Specific objectives

To describe the sociodemographic characteristics of children and adolescents with chronic kidney disease undergoing HD and their family caregivers, and to verify and compare the seroprevalence of SARS-CoV-2 in children and adolescents undergoing HD and their family caregivers.

## METHODS

### Ethical aspects

The study was conducted in accordance with national and international ethics guidelines, and was approved by the *Universidade Federal de São Paulo* (UNIFESP) Research Ethics Committee.

Consent was obtained from all individuals involved in the study through the signing of the Informed Consent Form by parents or guardians over 18 years of age and by patients aged 14 to under 18 years (second copy). Children aged 6 to 14 years indicated on the Informed Assent Form whether or not they agreed to participate in the study.

### Study design

This is a cross-sectional and descriptive study that followed the recommendations of the STrengthening the Reporting of OBservational studies in Epidemiology(14) guidelines for observational studies in epidemiology.

### Study place and period

The study was developed in the *Hospital São Paulo* (HSP) Pediatric Nephrology Outpatient Clinic, a university hospital of UNIFESP. Collections were carried out from May to September 2021, before the COVID-19 vaccination period for the study population.

### Study participants

This was a convenience sample composed of ten individuals aged 0 to 18 years with end-stage renal disease undergoing HD and their respective guardians/family caregivers. Inclusion criteria were age under 18 years, being under HD in the HSP Pediatric Nephrology Outpatient Clinic, a university hospital of UNIFESP, and performing the collection of biological material proposed in the study design. A person who accompanies a patient during dialysis sessions and who lives in the same household, being responsible for home care for this patient, was accepted as a companion/family caregiver. Exclusion criteria were being on another RRT method and declining consent during the data collection period.

### Procedures

Blood samples were obtained by peripheral venipuncture or from HD access, strictly following asepsis criteria and HSP protocols for such procedures. The sample was collected in a tube with clot activator, homogenized and left to rest at a controlled temperature between 2°C and 8°C. The tube was then sent to the HSP clinical analysis laboratory for analysis. Serological assessment for COVID-19 was performed using an anti-SARS-CoV-2 immunoassay for the quantitative determination of antibodies against the SARS-CoV-2 spike protein (S) receptor-binding domain.

Data related to sociodemographic characteristics were obtained from medical records using a semi-structured instrument developed by the researchers based on a literature review. The characteristics assessed were biological sex, age, ethnicity/race, school year, education level of companion/guardian, family income, receipt of sickness benefit or government benefit, means of transportation to go to the unit and mean travel time, type of residence, residence characteristics, number of rooms in the residence, number of people living in the residence, and whether there is running water and a sewage system in the residence.

### Statistical analysis

The collected data and results obtained were organized in a database and submitted to descriptive statistical analysis, with measures of frequency, central tendency (mean and median) and measures of dispersion (standard deviation). To test the association between two variables, the outcome (positive/negative serology) and sociodemographic characteristics, Fisher’s exact test was used with a significance level of 0.05%.

## RESULTS

Ten patients and their companions/family caregivers were included in the study. All patients underwent three weekly HD sessions lasting an average of four hours. At the time of collection, none of the participants had received the COVID-19 vaccine, and all denied presenting flu-like signs and symptoms at the time of collection and in the week prior to collection.

### Sociodemographic data

The sociodemographic data described in Table 1 demonstrate that the mean age was 11.47 years ± 4.11 (11 years and 5 months). Biological sex was 70% (7/10) male and 30% (3/10) female; selfdeclared ethnicity was 60% (6/10) mixed race and 40% (4/10) white; education level was 10% (1/10) for preschool, 80% (8/10) for incomplete elementary school and 10% (1/10) for incomplete high school. Among family companions/caregivers, 100% (10/10) are female, with an average age of 38.41 years (38 years and 4 months), with 20% (2/11) having incomplete elementary education, 20% (2/10) having incomplete high school education, and 60% (6/10) having completed high school education.

**Table 1:** Sociodemographic characteristics of children and adolescents with chronic kidney disease under outpatient hemodialysis, São Paulo, São Paulo, Brazil, 2021 (N=10)

Characteristics	n	Percentage %	Standard deviation
**Sex**			
Female	3	30	
Male	7	70	
**Age (years)**			
Minimum	5.46		
Mean	11.47		± 4.11
Maximum	16.0		
**Skin color/ethnicity**			
White	4	40	
Mixed race	6	60	
**Education**			
Preschool	2	20	
Incomplete elementary school	5	50	
Complete elementary school	0	0	
Incomplete high school	3	30	
Complete high school	0	0	
**Total family income**			
Up to 1 minimum wage	3	30	
1-3 minimum wages	5	50	
3-5 minimum wages	1	10	
More than 5 minimum wages	1	10	
**Per capita income in minimum wage**			
Up to 0.5 minimum wage (R$550.00)	3	30	
From 0.5 minimum wage to 1 minimum wage (R$550.00 to R$1,100)	6	60	
Greater than 1 minimum wage (R$1,100)	1	10	
Do you receive any type of benefit/assistance/pension?*			
Yes	3	30	
No	7	70	
**Means of transport**			
Car	2	20	
Car, bus, train, subway	1	10	
Bus, train, subway	4	40	
Ambulance	2	20	
Ambulance, train, subway	1	10	
**Travel time (hours)**			
Minimum	1.5		
Mean	2.15		± 1.13
Maximum	3.5		
**Residence**			
Owned	4	40	
Rented	4	40	
Granted	2	20	
**Residence characteristic**			
Masonry	10	100	
**Number of rooms in the residence**			
Minimum	3.0		
Mean	4.0		± 1.82
Maximum	6.0		
**Number of residents**			
Minimum	2.0		
Mean	3.3		± 1.82
Maximum	6.0		
**Sewage network**			
Yes	10	100	
No	0	0	
**Running water**			
Yes	10	100	
No	0	0	

n – absolute number; ±standard deviation; *Benefit, assistance and/or pension offered by the Brazilian National Social Security Institute.

Considering that the current minimum wage for the year 2021 was R$1,100.00, family income in minimum wage (MW) was 30% (3/10) up to 1 MW, 50% (5/10) from 1 to 3 MWs and 20% (2/10) above 3 MWs. It was included in the income that 30% (3/10) children received some type of government benefit or sickness benefit, the value of which depends on the agency and type of assistance. In relation to the number of residents in the family residence, including the child, 50% (5/10) represented 3, 30% (3/10) represented 4 and 20% (2/10) represented 5 or more individuals. Thus, the *per capita* income in MW was 30% (3/10) up to 0.5 MW (up to R$ 550.00), 20% (2/10) up to 0.75 MW (up to R$ 850.00), 30% (3/11) up to 1 MW (up to R$ 1,100.00) and 10% (1/10) greater than 1 MW *per capita* (above R$ 1,100.00) (Table 1).

Regarding the characteristics of the residence in which children or adolescents live, 100% (10/10) are made of masonry and have basic sanitation conditions with running water and a sewage system. Of these, 40% (4/10) are owned; 40% (4/10) are rented; and 20% (2/10) are provided. As for the number of rooms, 20% (2/10) have 3, 20% (2/10) have 4, 40% (4/10) have 5, and 20% (2/10) have 6 or more (Table 1).

Concerning the characteristics of the residence in which children or adolescents live, 100% (10/10) are made of masonry and have basic sanitation conditions with running water and a sewage system. Of these, 40% (4/10) are owned; 40% (4/10) are rented; and 20% (2/10) are provided. Regarding the number of rooms, 20% (2/10) have 3, 20% (2/10) have 4, 40% (4/10) have 5, and 20% (2/10) have 6 or more (Table 1).

### COVID-19 serology

Anti-SARS-CoV-2 serologies were performed by immunoassay for quantitative determination of antibodies against the SARS-CoV-2 S protein receptor binding domain. Values greater than or equal to 0.8 U/mL were considered reactive, and values below 0.8 U/mL were considered non-reactive. Among children and adolescents, eight (80%) presented non-reactive results with values below 0.4 U/mL, and two (20%) presented reactive results with values of 25.2 and above 250 U/mL. Among companions/family caregivers, there were seven (70%) non-reactive serological results below 0.4 U/mL and three (30%) reactive results with U/mL values of 31.5, 79.9 and 2.1, respectively (Table 2). COVID-19 seroprevalence was identified in children and adolescents undergoing HD therapy of 20% (2/10), in family companions, of 30% (3/10), and in the total group, of 25% (5/20).

**Table 2:** Results of serological tests for SARS-CoV-2 in children and adolescents with chronic kidney disease under hemodialysis and their companions/family caregivers, São Paulo, São Paulo, Brazil, 2021 (N=20)

Serological test	Patients (10)		Companions (10)	
	n	%	n	%
Reagent	2	20	3	30
Non-reactive*	8	80	7	70

n – absolute value; % – percentage; *Values lower than 0.8 U/mL are considered non-reactive.

The results matched between child or adolescent and their family companion in only one of the reactive cases (10%). On the other hand, there was one seropositive patient, without correlation with their guardian, and two reactive companions, with their respective non-reactive minors.

In bivariate analysis, considering the result of the serological test for SARS-CoV-2 and sociodemographic variables, no statistically significant association was identified for either patients (Table 3) or companions/family caregivers (Table 4).

**Table 3:** Relationship between SARS-CoV-2 serology and sociodemographic characteristics of children and adolescents with chronic kidney disease under outpatient hemodialysis, São Paulo, São Paulo, Brazil, 2021 (N=10)

Characteristics	SARS-CoV-2 serology		*p* value^#^
	Positive (%)	Negative (%)	
**Sex**			
Male	2 (100)	5(62.5)	1
Female	0	3(37.5)	
**Ethnicity**			
White	1(50)	3(37.5)	1
Mixed race	1(50)	5(62.5)	
**Education**			
Incomplete elementary school	2(100)	6(75)	1
Incomplete high school	0	1(12.5)	
Does not attend regular school	0	1(12.5)	
**Family income**			
Up to 1 minimum wage	1(50)	2(25)	1
From 1 to 3 minimum wages	1(50)	4(50)	
From 3 to 5 minimum wages	0	1(12.5)	
More than 5 minimum wages	0	1(12.5)	
Do you receive any type of benefit/assistance/pension?*			
Yes	1(50)	2(25)	1
No	1(50)	6(75)	
**Means of transport**			
Car	0	2(25)	0.511
Car, bus, train, subway	0	1(12.5)	
Bus, train, subway	1(50)	3(37.5)	
Ambulance	0	2(25)	
Ambulance, train, subway	1(50)	0	
**Residence**			
Owned	2 (100)	4 (50)	0.289
Leased		2 (250	
Granted		2(25)	
**Flu-like symptoms**			
Did not present	2 (100)	8 (100)

#Fisher’s exact test; *Benefit, assistance and/or pension offered by the Brazilian National Social Security Institute.

**Table 4:** Relationship between SARS-CoV-2 serology and sociodemographic characteristics of family caregivers of children and adolescents with chronic kidney disease under outpatient hemodialysis, São Paulo, São Paulo, Brazil, 2021 (N=10)

Characteristic	SARS-CoV-2 serology		*p* value^#^
	Positive (%)	Negative (%)	
**Sex**			
Female	3 (100)	7(700)	1
**Education**			
Incomplete elementary school	1 (33.3)	1 (14.3)	1
Incomplete high school	0	2 (28.6)	
Complete high school	2 (66.7)	4 (57.1)	
**Flu-like symptoms**			
Did not present	3 (100)	7 (100)	1

*^#^Fisher’s exact test.*

## DISCUSSION

The results of this study highlight not only the importance of screening for COVID-19 seroprevalence in children and adolescents undergoing HD during the period of major social restrictions of the pandemic, but also the relevance of the SDGs to address public health and well-being issues(12,13). It is noteworthy that to date this is the only study that includes family caregivers in screening for COVID seroprevalence in children and adolescents under HD.

The evidence from this study indicated that there were two patients (20%) and three companions (30%) with a reactive result in the serological test, i.e., 25% of the total group (patients and companions). Therefore, these five individuals had contact with and developed SARS-CoV-2 infection and did not present symptoms, meaning that they had an asymptomatic case of COVID-19.

A similar study conducted with pediatric nephrology centers in Istanbul, Turkey, found that the majority (88.2% 15/17) of patients with chronic kidney disease on dialysis who tested positive for SARS-CoV-2 were asymptomatic or had mild symptoms; however, the other two children in this group had severe symptoms and one of them died(15).

An European cohort of 582 children and adolescents found pediatric mortality rate for COVID-19 to be around 0.7%(16). UK records suggest this figure rises to 3.5% in children with chronic kidney disease and 16.2% in those under HD(17). In Egypt, a study concluded that children under HD have higher risks and worse prognoses for COVID-19 than children who have undergone kidney transplants(11).

The seropositive results for SARS-CoV-2 matched in only one of the pairs (10%), while non-reactive results were observed in 60% of cases, totaling 70% serological coincidence. The proposal to include family companions/caregivers was precisely to assess this relationship, since there is a high level of contact and proximity between the pair. In addition to this, guardians attend the same environments as children, including HD sessions. Several studies on COVID-19 in children also indicate that the infection occurred, mostly, after close contact with family members and people with confirmed COVID-19(4,8,9,10).

Therefore, even though the majority of the pediatric population does not have as high an incidence and mortality rate for COVID-19 as adults, the group affected by chronic kidney disease has higher mortality and severity than healthy children. The risk of infection is also increased, given the frequent trips to and from hospitals and healthcare services, contact with other patients and healthcare professionals, and the fact that the non-hospital dialysis environment itself (centers and outpatient clinics) is shared and has a high flow of people(18).

The COVID-19 pandemic has brought a series of difficulties and challenges to everyone, and has exacerbated social and economic inequalities, since although it has affected the entire globe, the consequences have been much more severe for the most vulnerable groups(19). This global crisis has highlighted health inequities between highand low-income groups and locations, such as precarious, unprepared, insufficient health systems and those lacking the financial, material and professional resources to respond to the overwhelming waves of SARS-CoV-2 infections(20). One of the greatest difficulties imposed by the COVID-19 pandemic for children with chronic kidney disease under HD was therapeutic routine continuity, such as HD sessions performed three times a week, examinations, consultations, procedures and hospital admissions(21).

In addition to the therapeutic routine, the possibility of moving around was another factor that was greatly compromised by non-pharmacological measures to protect against and prevent the virus. In the city of São Paulo, where this study was conducted, as well as in several regions of the state, quarantine or “lockdown” protocols, isolation and social distancing were instituted(22,23). These measures could not be adopted by children under HD and their families, due to the need to travel to undergo vital treatment(11,24).

Furthermore, 40% of participants used public transportation exclusively as a means of transportation and spent a considerable amount of time commuting (more than two hours, on average), increasing the risk of exposure to SARS-CoV-2. This situation directly impacted adherence to dialysis, according to a study that mapped the inequities faced in HD treatment during the COVID-19 pandemic. Data from different countries were analyzed, identifying an increase of 64% and 67% in the number of missed HD sessions during the pandemic in lowand middle-income countries, respectively(21).

Furthermore, socioeconomic vulnerability may have been aggravated during the pandemic, when unemployment and inequality levels increased. A study based on the “ConVid Behavior Survey” questionnaire, conducted by the Oswaldo Cruz Foundation with 45,161 validated responses, identified that there was a 62.2% decrease in family income, and it was also found that the higher the family income, the smaller the decrease in income(25), i.e., low-income people suffered even more from the financial impacts of the pandemic.

The Brazilian Federal Government identifies socioeconomically vulnerable families for inclusion in social assistance and income redistribution programs through the Single Registry. This instrument classifies as “low-income families” those with a monthly *per capita* income of up to half the minimum wage (R$550.00 in 2021) or that earn a total of up to three minimum wages per month (R$3,300.00 in 2021). Based on the sociodemographic data collected in this study, it can be inferred that 80% (8/10) of family groups are low-income, with 30% of participants receiving some type of government financial subsidy(26,27).

Poverty compromises access, pursuit and quality of health, and adherence to treatments, and is associated with greater environmental exposure and risk behaviors, in a way that drastically increases socioeconomic disparities and impacts all areas of life(28). Even though treatments are offered free of charge by the Brazilian Health System, the routine of treatments generates expenses for transportation, food and extras for the family. There is also the time required for child healthcare, which in this population is full-time due to the necessary medications and assistance. This prevents their main caregivers from taking on formal work, further damaging the family income. This situation directly impacts the quality of life of children and their families, making it difficult to adhere to treatment and other areas of life, such as education and leisure.

It is also observed that all children and adolescents live in masonry homes with a basic sanitation system (piped water and sewage) in the Metropolitan Region of the state of São Paulo. Thus, even though they present many socioeconomic vulnerabilities, they are established in the Brazilian region with the best Human Development Index, economic development and largest network of public healthcare services, factors that contribute positively to access to quality healthcare and meeting the specific demands and treatments that they have(29,30).

The 17 SDGs are global goals set by the UN by 2030 to eradicate poverty, protect the planet and ensure peace and prosperity for all people(18). In view of this, Eradicate Poverty and Reduce Inequalities, SDGs 1 and 10, respectively, even if they do not refer to health directly, are intrinsically connected with the provision of equitable assistance and care. Combating inequities and poverty and promoting social inclusion generate better living conditions and, consequently, better health(28,30).

SDG 3, Good Health and Well-being, which aims to provide access to quality healthcare and promote well-being for all, does not propose actions that directly address issues related to chronic kidney disease and HD. However, these actions are mainly aimed at primary healthcare, where there is great potential for preventing, diagnosing and treating chronic kidney disease early, potentially preventing its progression to dialysis stages(30).

Furthermore, one of the goals established in SDG 3 is to reduce premature mortality from non-communicable diseases, through prevention and early treatment. However, the UN focused SDG 3 on mental health and four types of chronic diseases: cardiovascular; respiratory; cancer; and diabetes. Thus, kidney disease was neglected in this planning, despite being one of the main and fastest growing causes of morbidity and mortality in the world. This fact illustrates and supports the inequity and difficulty in accessing RRT, due to the global lack of effective health programs and policies(28,31,32,33).

Therefore, the aforementioned SDGs are directly and indirectly related to the demands of the child population with chronic kidney disease. Our results corroborate numerous national and international studies that demonstrate the social vulnerability, socioeconomic inequities and health needs of this group as well as the effects on quality of life resulting from health-disease conditions and life-sustaining therapies required by this population.

### Study limitations

The limitations of this study are related to a restricted sample, which can be justified by the fact that it only included one dialysis center with limited and small service capacity. However, this characteristic is common to pediatric dialysis services, since there is another treatment method, PD, and this population is fluctuating, as it is a priority for transplantation. These limitations are also observed in multiple international studies, such as those referenced, about this population and topic. Moreover, the sociodemographic profile is specific and regionalized in a way that does not represent the multiple Brazilian realities.

### Contributions to nursing, health or public policy

This study presents a vast wealth of data regarding sociodemographic and epidemiological issues of the population studied. In addition, it is part of a macro-study that collects these and other data in the child and adolescent population that underwent RRT during the pandemic. Such a large amount and wealth of information can result in several products and contribute significantly to scientific production in health on this specific and relevant subject, in addition to encouraging further studies and guiding healthcare practice.

## CONCLUSIONS

COVID-19 seroprevalence was identified in children and adolescents under HD and in their companions/family caregivers. An important characteristic identified among seroreactive individuals is the asymptomatic presentation of the disease. The sociodemographic variables studied demonstrated the vulnerability of this population, which, added to inequities in access to effective treatment for kidney disease, compromise child and family quality of life, including their survival.

Guided by the SDGs, robust national and international public policies are needed, encouraged by UN agencies. These actions must be multisectoral and strategic, ranging from the prevention of diseases that affect the kidneys to renal replacement therapies, to eradicate social and health inequities in this population, ensuring access to and quality of care.

Finally, further studies on the viral dynamics of SARS-CoV-2 in children under HD are needed to elucidate the clinical and epidemiological variables involved, in addition to studies with a wider range of participants, dialysis centers and regions to reliably demonstrate the reality of pediatric HD in Brazil and worldwide.

## Data Availability

The research data are available only upon request.
