# Supplementary material for: Sociodemographic Profile of Pediatric Hemodialysis during the COVID-19 Pandemic and Challenges for Sustainable Development
Source: Rev Bras Enferm. 2025 Oct 27;78(Suppl 1):e20240041. doi: 10.1590/0034-7167-2024-0041 (PMC12563836; doi:10.1590/0034-7167-2024-0041)
Supplement: Translation

# Translation: Perfil Sociodemográfico da Hemodiálise Pediátrica na Pandemia de COVID-19 e Desafios para o Desenvolvimento Sustentável

**DOI:** 10.1590/0034-7167-2024-0041pt

**Authors:** Nathália Cardoso Neves, Richarlisson Borges de Morais, Isis Oliveira Arruda, Maria Luiza Dautro Moreira do Val, Ana Lúcia Cardoso Santos Abreu, Maria Cristina de Andrade, Paulo Henrique Braz-Silva, Monica Taminato

**Affiliations:** I Universidade Federal de São Paulo. São Paulo, São Paulo, Brasil; II Universidade Federal de Uberlândia. Uberlândia, Minas Gerais, Brasil; III Universidade Federal de São Paulo, Hospital São Paulo. São Paulo, São Paulo, Brasil; IV Universidade de São Paulo. São Paulo, São Paulo, Brasil

**Keywords:** Pediatria, Hemodiálise, COVID-19, Fatores Demográficos, Objetivos de Desenvolvimento Sustentável

## Abstract

**Objetivos:**

relacionar as características sociodemográficas com a sorologia para SARS-CoV-2 de menores em hemodiálise e seu cuidador.

**Métodos:**

estudo transversal, descritivo, com análise de imunoensaio anti-SARS-CoV-2 Spike.

**Resultados:**

foram incluídos dez pacientes e seus acompanhantes, antes da vacinação contra COVID-19. A soroprevalência global foi de 25% e 20% entre os pacientes, os quais tiveram média de idade de 11,47 anos, sendo 70% do sexo masculino. Todos os acompanhantes eram mulheres (100%), com idade média de 38,41 anos e 60% de etnia parda. Metade (50%) recebe de 1 a 3 salários mínimos, e 70% gastam mais de duas horas de deslocamento. Quanto à residência, todas (100%) são de alvenaria e com saneamento básico.

**Conclusões:**

evidencia-se a importância do rastreamento de casos de COVID-19 como rotina e o impacto das variáveis socioeconômicas na qualidade de vida das crianças e familiares.

## INTRODUÇÃO

A doença renal crônica é um problema de saúde pública que apresenta grande aumento de incidência e prevalência em todo o mundo, e está associada a maior morbimortalidade. É caracterizada pela perda gradual, progressiva e irreversível da função renal, sobretudo da capacidade de filtração glomerular. A evolução do quadro leva à falência renal e à necessidade de Terapia Renal Substitutiva (TRS), sendo atualmente disponíveis as diálises (hemodiálise (HD) e diálise peritoneal (DP)) e transplante renal(1,2).

Embora os métodos de TRS ofereçam um grande aumento da sobrevida, podem apresentar também complicações metabólicas, cardiovasculares e infecciosas, além de comprometerem, no público infantil, o crescimento, desenvolvimento e qualidade de vida das crianças e suas famílias. Estudo epidemiológico que reuniu dados globais indica que as crianças submetidas à HD têm taxas de mortalidade 30 vezes maiores que crianças saudáveis, tendo como principais causas doenças cardiovasculares (30% a 40%) e infecções (20% a 50%)(3). Outro estudo revela que essas taxas de mortalidade podem aumentar de 30 a 150 vezes e que a expectativa de vida é de aproximadamente 50 anos a menos do que a população pediátrica sem comorbidades(2).

Com o advento da emergência pandêmica da COVID-19, declarada em março de 2020 pela Organização Mundial da Saúde, os desafios enfrentados pelas crianças e adolescentes em tratamento hemodialítico se tornaram ainda mais complexos. Essa nova doença é causada pelo vírus SARS-CoV-2, da família *Coronaviridae*, transmitida por aerossóis e objetos contaminados, causando a Síndrome Respiratória Aguda Grave por SARS-CoV-2(4,5,6,7).

Revisões de literatura indicam que as crianças apresentam um bom prognóstico para a doença, com recuperação em duas semanas e sintomatologia de leve a moderada, sendo mais frequentes sintomas como febre, tosse seca, fadiga, congestão nasal e coriza. Além disso, indicam que a maioria das transmissões ocorreu por contato próximo com familiares ou pessoas com diagnóstico positivo para COVID-19(4,8,9,10). Estudo pediátrico que incluiu dados da China, Itália e Estados Unidos demonstrou que as crianças representam uma variação de 1% a 5% dos casos confirmados de COVID-19, sendo 2% dos casos na China (4.672), 1,2%, na Itália (22.500), e 5%, nos Estados Unidos (4.226). Desse montante, identificaram-se apenas dois casos de óbito em crianças com diagnóstico de COVID-19(8).

Já em relação às crianças com doença renal crônica, estudo egípcio apontou taxa de incidência de 16,2% de COVID-19 entre as submetidas à HD e taxa de mortalidade de 15,7% naquelas em TRS. O estudo atribui essa incidência ao fato que essa população é impossibilitada de cumprir uma das principais medidas de prevenção à exposição ao vírus SARS-CoV-2, o distanciamento social, haja vista os deslocamentos para as sessões de HD múltiplas vezes por semana, além das rotinas de consultas e exames de acompanhamento de saúde(11).

Os 17 Objetivos do Desenvolvimento Sustentável (ODS) são metas globais estabelecidas pela Organização das Nações Unidas (ONU) até 2030 para erradicar a pobreza, proteger o planeta e garantir a paz e prosperidade a todas as pessoas(12). O acesso a serviços de saúde de qualidade, como a HD, desempenha um papel crucial no alcance do ODS 3 – Saúde e Bem-Estar.

Além disso, o suporte e a educação fornecidos às famílias dessas crianças desempenham um papel fundamental, ajudando a mitigar o estresse emocional e financeiro associado ao tratamento de longo prazo. Garantir o acesso equitativo a serviços de saúde de qualidade para todas as crianças, independentemente de sua condição médica ou localização geográfica, é essencial para promover não apenas sua saúde, mas também a sustentabilidade das comunidades e sociedades como um todo(13).

Diante dos ODS estabelecidos pela ONU, é evidente que as crianças submetidas à HD constituem um grupo vulnerável, não apenas devido a questões de saúde, mas também aos desafios sociodemográficos e econômicos relacionados à vida cotidiana. Dessa forma, também foi incluído no rastreio o seu acompanhante/cuidador familiar, tendo em vista que o ambiente da HD ambulatorial é compartilhado e que há grande proximidade e convivência desse agente no cuidado das crianças dessa população. Nessa perspectiva, torna-se fundamental investigar a ocorrência de COVID-19 e as variáveis sociodemográficas que impactam a qualidade de vida e o acesso à saúde desse grupo. Dessa forma, o conhecimento científico poderá fundamentar as estratégias e ações que conduzam aos objetivos de Erradicação da Pobreza (ODS 1) Saúde de Qualidade (ODS 3) e Redução das Desigualdades (ODS 10), proporcionando uma existência digna a essa população(13).

## OBJETIVOS

### Objetivo geral

Analisar as características sociodemográficas dos pacientes em HD pediátrica e seu cuidador familiar e relacionar com a sorologia para SARS-CoV-2.

### Objetivos específicos

Descrever as características sociodemográficas em crianças e adolescentes com doença renal crônica em tratamento hemodialítico e seu cuidador familiar, e verificar e comparar a soroprevalência para SARS-CoV-2 em crianças e adolescentes em HD e seu cuidador familiar.

## MÉTODOS

### Aspectos éticos

O estudo foi conduzido de acordo com as diretrizes de ética nacionais e internacionais, sendo aprovado pelo Comitê de Ética em Pesquisa com Seres Humanos da Universidade Federal de São Paulo (UNIFESP).

O consentimento foi obtido de todos os indivíduos envolvidos no estudo por meio da assinatura do Termo de Consentimento Livre e Esclarecido pelos pais ou responsáveis maiores de 18 anos e pelos pacientes da faixa etária de 14 anos a 18 anos incompletos (segunda via). As crianças de 6 a 14 anos indicaram no Termo de Assentimento Livre e Esclarecido se aceitavam ou não participar do estudo.

### Tipo de estudo

Estudo transversal e descritivo que seguiu as recomendações das diretrizes *STrengthening the Reporting of OBservational studies in Epidemiology*(14) para estudos observacionais em epidemiologia.

### Local e período do estudo

O estudo foi desenvolvido no Ambulatório de Nefrologia Pediátrica do Hospital São Paulo (HSP), hospital universitário da UNIFESP. As coletas foram realizadas no período de maio a setembro de 2021, antes do período de vacinação contra COVID-19 para a população em estudo.

### Participantes do estudo

Trata-se de amostra por conveniência composta por dez indivíduos de 0 a 18 anos com doença renal crônica terminal em TRS hemodialítica e seus respectivos responsáveis acompanhantes/cuidador familiar. Os critérios de inclusão foram idade inferior a 18 anos, estar em HD no Ambulatório de Nefrologia Pediátrica do HSP, hospital universitário da UNIFESP, e realizar a coleta de material biológico proposta no desenho do estudo. Admitiu-se como acompanhante/cuidador familiar a pessoa que acompanha o paciente nas sessões de diálise e que reside no mesmo domicílio, responsabilizando-se pelos cuidados domiciliares com o mesmo. Os critérios de exclusão foram estar sob outro método de TRS e declínio do consentimento durante o período de coleta de dados.

### Procedimentos

As amostras de sangue foram obtidas por punção venosa periférica ou do acesso para HD seguindo rigorosamente os critérios de assepsia e os protocolos do HSP para tais procedimentos. A amostra foi coletada em tubo com ativador de coágulo, homogeneizado e deixado em repouso em temperatura controlada entre 2°C e 8°C. Em seguida, o tubo foi encaminhado ao laboratório de análises clínicas do HSP para análise. A avaliação sorológica para COVID-19 foi realizada através de imunoensaio anti-SARS-CoV-2 para a determinação quantitativa de anticorpos contra o domínio de ligação ao receptor da proteína spike (S) do SARS-CoV-2.

Os dados relacionados às características sociodemográficas foram obtidos nos prontuários utilizando instrumento semiestruturado elaborado pelos pesquisadores a partir da revisão da literatura. As características avaliadas foram sexo biológico, idade, etnia/raça, ano escolar, escolaridade do acompanhante/responsável, renda familiar, recebimento de auxílio-doença ou benefício governamental, meio de transporte para se deslocar até a unidade e o tempo médio de deslocamento, tipo de residência, características da residência, número de cômodos da residência, quantidade de pessoas que moram na residência, e se há água encanada e rede de esgoto na moradia.

### Análise estatística

Os dados coletados e resultados obtidos foram organizados em um banco de dados e submetidos à análise estatística descritiva, com medidas de frequência, tendência central (média e mediana) e medidas de dispersão (desvio padrão). Para testar a associação entre duas variáveis, o desfecho (sorologia positiva/negativa) e as características sociodemográficas, utilizou-se o teste exato de Fisher com nível de significância de 0,05%.

## RESULTADOS

Foram incluídos no estudo dez pacientes e seus acompanhantes/cuidadores familiares. Todos os pacientes realizavam três sessões de HD semanais com duração média de quatro horas. No momento da coleta, nenhum dos participantes havia recebido a vacina contra COVID-19, e todos negaram apresentar sinais e sintomas gripais no ato e na semana anterior à coleta.

### Dados sociodemográficos

Os dados sociodemográficos descritos na Tabela 1 demonstram que a idade média foi de 11,47 anos ± 4,11 (11 anos e 5 meses). O sexo biológico foi 70% (7/10) masculino e 30% (3/10) feminino; a etnia autodeclarada foi 60% (6/10) parda e 40% (4/10) branca; a escolaridade foi 10% (1/10) para pré-escola, 80% (8/10) para ensino fundamental incompleto e 10% (1/10) para ensino médio incompleto. Já entre os acompanhantes/cuidadores familiares, 100% (10/10) são do sexo feminino, com idade média de 38,41 anos (38 anos e 4 meses), com 20% (2/11) tendo ensino fundamental incompleto, 20% (2/10), ensino médio incompleto, e 60% (6/10), ensino médio completo.

**Tabela 1:** Características sociodemográficas de crianças e adolescentes com doença renal crônica em hemodiálise ambulatorial, São Paulo, São Paulo, Brasil, 2021 (N=10)

Características	n	Porcentagem %	Desvio padrão
**Sexo**			
Feminino	3	30	
Masculino	7	70	
**Idade (anos)**			
Mínima	5,46		
Média	11,47		± 4,11
Máxima	16,0		
**Cor da pele/etnia**			
Branca	4	40	
Parda	6	60	
**Escolaridade**			
Pré-escola	2	20	
Fundamental incompleto	5	50	
Fundamental completo	0	0	
Ensino médio incompleto	3	30	
Ensino médio completo	0	0	
**Renda familiar total**			
Até 1 salário mínimo	3	30	
1-3 salários mínimos	5	50	
3-5 salários mínimos	1	10	
Superior a 5 salários	1	10	
**Renda *per capita* em salário mínimo**			
Até 0,5 salário mínimo (R$ 550,00)	3	30	
De 0,5 salário mínimo a 1 salário mínimo (R$ 550,00 a R$ 1.100)	6	60	
Maior que 1 salário mínimo (R$ 1.100)	1	10	
Recebe algum tipo de benefício/auxílio/pensão*			
Sim	3	30	
Não	7	70	
**Meio de transporte**			
Carro	2	20	
Carro, ônibus, trem, metrô	1	10	
Ônibus, trem, metrô	4	40	
Ambulância	2	20	
Ambulância, trem, metrô	1	10	
**Tempo de deslocamento (horas)**			
Mínima	1,5		
Média	2,15		± 1,13
Máxima	3,5		
**Residência**			
Própria	4	40	
Alugada	4	40	
Cedida	2	20	
**Característica da residência**			
Alvenaria	10	100	
**Nº de cômodos da residência**			
Mínima	3,0		
Média	4,0		± 1,82
Máxima	6,0		
**Nº de moradores**			
Mínima	2,0		
Média	3,3		± 1,82
Máxima	6,0		
**Rede de esgoto**			
Sim	10	100	
Não	0	0	
**Água encanada**			
Sim	10	100	
Não	0	0	

n – número absoluto; ±desvio padrão; *Benefício, auxílio e/ou pensão oferecidos pelo Instituto Nacional do Seguro Social.

Considerando que o salário mínimo vigente no ano de 2021 era de R$ 1.100,00, a renda familiar em salário mínimo (SM) foi de 30% (3/10) de até 1 SM, 50% (5/10) de 1 a 3 SMs e 20% (2/10) acima de 3 SMs. Foi incluído na renda que 30% (3/10) crianças recebiam algum tipo de benefício ou auxílio doença governamental, cujo valor depende do órgão e tipo de assistência. Em relação ao número de moradores na residência familiar, incluindo a criança, 50% (5/10) representava 3, 30% (3/10) representava 4 e 20% (2/10) representava 5 ou mais indivíduos. Assim, a renda *per capita* em SM foi de 30% (3/10) até 0,5 SM (até R$ 550,00), 20% (2/10) até 0,75 SM (até R$ 850,00), 30% (3/11) até 1 SM (até R$ 1.100,00) e 10% (1/10) maior que 1 SM *per capita* (acima de R$ 1.100,00) (Tabela 1).

Em relação às características da residência em que a criança ou adolescente vive, 100% (10/10) são de alvenaria e têm condições de saneamento básico com água encanada e rede de esgoto. Dessas, 40% (4/10) são próprias; 40% (4/10) são alugadas; e 20% (2/10) são cedidas. Quanto à quantidade de cômodos, 20% (2/10) apresentam 3, 20% (2/10), 4, 40% (4/10), 5, e 20% (2/10) apresentam 6 ou mais (Tabela 1).

Para se deslocar até o ambulatório de nefrologia pediátrica onde são realizadas as sessões de HD três vezes na semana, localizado na Vila Clementino, na cidade de São Paulo, 40% (4/10) das famílias (criança e acompanhante) utilizam transporte público coletivo (metrô, trem e/ou ônibus); 30% (3/10) usam veículo particular; e 30% (3/10) usam ambulância. Em relação ao tempo total de deslocamento, considerando a ida ao serviço e o retorno à residência, 30% (3/10) levam até duas horas, 30% (3/10), de duas a três horas, e 40% (4/10), mais de três horas (Tabela 1).

### Sorologia de COVID-19

As sorologias anti-SARS-CoV-2 foram realizadas por imunoensaio de determinação quantitativa de anticorpos contra domínio de ligação ao receptor da proteína S do SARS-CoV-2. Consideraram-se reagentes valores maiores ou iguais a 0,8 U/mL, e não reagentes, os valores inferiores a 0,8 U/mL. Entre as crianças e adolescentes, oito (80%) apresentaram resultados não reagentes com valores inferiores a 0,4 U/mL, e dois (20%) apresentaram resultados reagentes com valores de 25,2 e superiores a 250 U/mL. Já entre os acompanhantes/cuidadores familiares, houve sete (70%) resultados sorológicos não reagentes inferiores 0,4 U/mL e três (30%) resultados reagentes com valores em U/mL de 31,5, 79,9 e 2,1, respectivamente (Tabela 2). Foi identificada a soroprevalência de COVID-19 nas crianças e adolescentes em terapia hemodialítica de 20% (2/10), nos acompanhantes familiares, de 30% (3/10), e no grupo total, de 25% (5/20).

**Tabela 2:** Resultados dos testes sorológicos para SARS-CoV-2 de crianças e adolescentes com doença renal crônica em hemodiálise e seus acompanhantes/cuidadores familiares, São Paulo, São Paulo, Brasil, 2021 (N=20)

Teste sorológico	Pacientes (10)		Acompanhantes (10)	
	n	%	n	%
Reagente	2	20	3	30
Não reagente*	8	80	7	70

n – valor absoluto; % – porcentagem; *Valores menores que 0,8 U/mL são considerados não reagentes.

A correspondência dos resultados entre a criança ou adolescente e seu acompanhante familiar só ocorreu em um dos casos reagentes (10%). Em contrapartida, houve um paciente sororreagente, sem a correlação com a sua responsável, e duas acompanhantes reagentes, com seus respectivos menores não reagentes.

Na análise bivariada, considerando o resultado do teste sorológico para SARS-CoV-2 e as variáveis sociodemográficas, não foi identificada associação estatisticamente significante tanto para os pacientes (Tabela 3) quanto para os acompanhantes/cuidadores familiares (Tabela 4).

**Tabela 3:** Relação entre a sorologia para SARS-CoV-2 e as características sociodemográficas de crianças e adolescentes com doença renal crônica em hemodiálise ambulatorial, São Paulo, São Paulo, Brasil, 2021 (N=10)

Características	Sorologia SARS-CoV-2		Valor de *p* ^#^
	Positivo (%)	Negativo (%)	
**Sexo**			
Masculino	2 (100)	5(62,5)	1
Feminino	0	3(37,5)	
**Etnia**			
Branca	1(50)	3(37,5)	1
Parda	1(50)	5(62,5)	
**Escolaridade**			
Fundamental incompleto	2(100)	6(75)	1
Ensino médio incompleto	0	1(12,5)	
Não frequenta escola regular	0	1(12,5)	
**Renda familiar**			
Até 1 salário mínimo	1(50)	2(25)	1
De 1 a 3 salários mínimos	1(50)	4(50)	
De 3 a 5 salários mínimos	0	1(12,5)	
Mais de 5 salários mínimos	0	1(12,5)	
Recebe algum tipo de benefício/auxílio/pensão*			
Sim	1(50)	2(25)	1
Não	1(50)	6(75)	
**Meio de transporte**			
Carro	0	2(25)	0,511
Carro, ônibus, trem, metrô	0	1(12,5)	
Ônibus, trem, metrô	1(50)	3(37,5)	
Ambulância	0	2(25)	
Ambulância, trem, metrô	1(50)	0	
**Residência**			
Própria	2 (100)	4 (50)	0,289
Alugada		2 (250	
Cedida		2(25)	
**Sintomas gripais**			
Não apresentava	2 (100)	8 (100)	

^#^Teste exato de Fisher; *Benefício, auxílio e/ou pensão oferecidos pelo Instituto Nacional do Seguro Social.

**Tabela 4:** Relação entre a sorologia para SARS-CoV-2 e as características sociodemográficas de acompanhantes/cuidadores familiares de crianças e adolescentes com doença renal crônica em hemodiálise ambulatorial, São Paulo, São Paulo, Brasil, 2021 (N=10)

Característica	Sorologia SARS-CoV-2		Valor de *p* ^#^
	Positivo (%)	Negativo (%)	
**Sexo**			
Feminino	3 (100)	7(700)	1
**Escolaridade**			
Fundamental incompleto	1 (33,3)	1 (14,3)	1
Ensino médio incompleto	0	2 (28,6)	
Ensino médio completo	2 (66,7)	4 (57,1)	
**Sintomas gripais**			
Não apresentava	3 (100)	7 (100)	1

*^#^Teste exato de Fisher.*

## DISCUSSÃO

Os resultados deste estudo destacam não apenas a importância do rastreio da soroprevalência de COVID-19 em crianças e adolescentes em HD no período de grandes restrições sociais da pandemia, mas também a relevância dos ODS para abordar questões de saúde pública e bem-estar(12,13). Destaca-se que, até o momento, este é o único estudo que inclui o cuidador familiar no rastreio da soroprevalência de COVID em crianças e adolescentes em HD.

As evidências deste estudo indicaram que havia dois pacientes (20%) e três acompanhantes (30%) com resultado reagente no teste sorológico, ou seja, 25% do grupo total (pacientes e acompanhantes). Por conseguinte, esses cinco indivíduos tiveram contato e desenvolveram a infecção por SARS-CoV-2 e não apresentaram sintomas, significando que tiveram um quadro assintomático de COVID-19.

Estudo semelhante realizado com centros de nefrologia pediátrica em Istambul, na Turquia, constatou que a maioria (88,2% 15/17) dos pacientes com doença renal crônica em tratamento dialítico que testou positivo para SARS-CoV-2 foi assintomática ou apresentou sintomas leves; contudo, as duas outras crianças desse grupo tiveram quadros severos e uma delas foi a óbito(15).

Coorte europeia com 582 crianças e adolescentes constatou o índice de mortalidade pediátrica para COVID-19 em cerca de 0,7%(16). Os registros do Reino Unido sugerem que essa estatística aumente para 3,5% em crianças com doença renal crônica e para 16,2% nas que realizam HD(17). Já no Egito, estudo concluiu que crianças em HD apresentam maiores riscos e piores prognósticos para COVID-19 do que crianças transplantadas renais(11).

A correspondência de resultados sororreagentes para SARS-CoV-2 ocorreu em apenas um dos binômios (10%), enquanto a de não reagentes foi observada em 60% dos casos, totalizando 70% de coincidência de sorologia. A proposta de incluir os acompanhantes/cuidadores familiares foi justamente para avaliar essa relação, uma vez que há um grande nível de contato e proximidade entre o binômio. Além disso, os responsáveis frequentam os mesmos ambientes que a criança, incluindo as sessões de HD. Diversos estudos acerca da COVID-19 em crianças também apontam que a infecção se deu, majoritariamente, após o contato próximo com membros da família e pessoas com COVID-19 confirmada(4,8,9,10).

Portanto, mesmo que a maioria da população pediátrica não apresente incidência e mortalidade para COVID-19 tão altas quanto adultos, o grupo acometido por doença renal crônica possui maior mortalidade e gravidade que crianças saudáveis. O risco de infecção também é aumentado, haja vista as idas e vindas frequentes dos ambientes hospitalares e serviços de saúde, contato com outros pacientes e profissionais de saúde e que o próprio ambiente não hospitalar de diálise (centros e ambulatórios) são compartilhados e possuem alto fluxo de pessoas(18).

A pandemia de COVID-19 trouxe uma série de dificuldades e desafios para todo mundo, e exacerbou as desigualdades sociais e econômicas, visto que embora tenha atingido todo o globo, as consequências foram muito mais severas aos grupos mais vulneráveis(19). Essa crise global evidenciou as iniquidades em saúde entre os locais e grupos de alta e baixa renda, como sistemas de saúde precários, despreparados, insuficientes e sem os recursos financeiros, materiais e profissionais para atender às avassaladoras ondas de infecções pelo SARS-CoV-2(20). Uma das maiores dificuldades imposta pela pandemia de COVID-19 para as crianças com doença renal crônica em HD foi a continuidade da rotina terapêutica, como sessões de HD realizadas três vezes por semana, exames, consultas, procedimentos e internações hospitalares(21).

Além da rotina terapêutica, a possibilidade de se locomover foi outro fator muito comprometido pelas medidas não farmacológicas de proteção e prevenção ao vírus. Na cidade de São Paulo, local onde foi realizado este estudo, assim como em diversas regiões do estado, foram instituídos protocolos de quarentena ou “*lockdown*”, isolamento e distanciamento social(22,23). Essas medidas não puderam ser adotadas pelas crianças em HD e suas famílias, devido à necessidade de se deslocarem para realizar um tratamento vital(11,24).

Ademais, 40% dos participantes utilizavam exclusivamente o transporte público coletivo como meio de locomoção e gastavam um tempo considerável para o deslocamento (superior a duas horas, em média), aumentando o risco de exposição ao SARS-CoV-2. Tal conjuntura impactou diretamente a adesão à diálise, conforme relato de estudo que mapeou as iniquidades enfrentadas no tratamento hemodialítico durante a pandemia de COVID-19. Foram analisados dados de diferentes países, identificando-se aumento de 64% e 67% no número de faltas em sessões de HD durante a pandemia em países de baixa e média renda, respectivamente(21).

Outrossim, a vulnerabilidade socioeconômica pode ter sido agravada durante a pandemia, em que houve o aumento nos níveis do desemprego e da desigualdade. Estudo baseado no questionário “ConVid Pesquisa de Comportamentos”, realizado pela Fundação Oswaldo Cruz com 45.161 respostas validadas, identificou que houve diminuição da renda familiar em 62,2%, constatando-se ainda que, quanto maior a renda familiar, menor a diminuição dos rendimentos(25), ou seja, pessoas de baixa renda sofreram ainda mais os impactos financeiros da pandemia.

O Governo Federal do Brasil realiza a identificação de famílias socioeconomicamente vulneráveis para inclusão em programas de assistência social e redistribuição de renda por meio do Cadastro Único. Esse instrumento classifica como “família de baixa renda” aquelas que apresentam renda mensal *per capita* de até meio salário mínimo (R$ 550,00 em 2021) ou que ganham no total de até três salários mínimos mensais (R$ 3.300,00 em 2021). Com base nos dados sociodemográficos levantados neste estudo, pode-se inferir que 80% (8/10) dos grupos familiares são de baixa renda, sendo que 30% dos participantes recebem algum tipo de subsídio financeiro governamental(26,27).

A pobreza compromete o acesso, a busca e qualidade da saúde, e adesão a tratamentos, e está associada a maior exposição ambiental e a comportamentos de risco, de forma que acentua drasticamente as disparidades socioeconômicas e impacta todos os âmbitos da vida(28). Mesmo que os tratamentos sejam ofertados de forma gratuita pelo Sistema Único de Saúde brasileiro, a rotina de tratamentos gera gastos com transporte, alimentação e extras para a família. Há, ainda, o tempo demandado para os cuidados de saúde da criança, que nesse público é integral devido às medicações e assistências necessárias. Isso impede que os seus principais cuidadores assumam um trabalho formal, prejudicando ainda mais a renda familiar. Tal conjuntura impacta diretamente a qualidade de vida das crianças e de seus familiares, dificultando a adesão ao tratamento e demais áreas da vida, como educação e lazer.

Observa-se também que todas as crianças e adolescentes moram em residências de alvenaria com sistema de saneamento básico (água e esgoto encanados) na Região Metropolitana do estado de São Paulo. Assim, mesmo que apresentem muitas vulnerabilidades socioeconômicas, estão estabelecidos na região brasileira com o melhor Índice de Desenvolvimento Humano, de desenvolvimento econômico e maior rede de serviços de saúde públicos, fatores que contribuem positivamente para o acesso à saúde de qualidade e atendimento às demandas e tratamentos específicos que possuem(29,30).

Os 17 ODS são metas globais estabelecidas pela ONU até 2030 para erradicar a pobreza, proteger o planeta e garantir a paz e prosperidade a todas as pessoas(18). À vista disso, Erradicação da Pobreza e Redução de Desigualdades, ODS 1 e 10, respectivamente, mesmo que não se refiram à saúde diretamente, estão conectadas intrinsecamente com a prestação de assistência e cuidados equitativos. O combate às iniquidades e pobreza e a promoção da inclusão social geram melhores condições de vida e, por conseguinte, de saúde(28,30).

O ODS 3, Saúde e Bem-Estar, que almeja o acesso à saúde de qualidade e a promoção do bem-estar a todas as pessoas, não propõe ações que atuam diretamente nas questões referentes à doença renal crônica e HD. Contudo, essas ações visam, principalmente, à Atenção Primária à Saúde, em que há grande potencial de prevenir, diagnosticar e tratar precocemente a doença renal crônica, podendo evitar sua progressão para estágios dialíticos(30).

Além disso, uma das metas estabelecidas no ODS 3 é a redução da mortalidade prematura por doenças não transmissíveis, atuando na prevenção e tratamento precoce. Entretanto, a ONU colocou o foco do ODS 3 na saúde mental e em quatro tipos de doenças crônicas: cardiovasculares; respiratórias; câncer; e diabetes. Assim, a doença renal foi negligenciada neste planejamento, apesar de ser uma das principais e mais crescentes causas de morbimortalidade no mundo. Tal fato ilustra e corrobora a iniquidade e dificuldade de acesso à TRS, devido à carência global de programas e políticas de saúde efetivas(28,31,32,33).

Portanto, os ODS mencionados estão relacionados, direta e indiretamente, às demandas da população infantil com doença renal crônica. Nossos resultados corroboram inúmeros estudos nacionais e internacionais que demonstram a vulnerabilidade social, as iniquidades socioeconômicas e as necessidades em saúde desse grupo, assim como os efeitos na qualidade de vida decorrentes de condições de saúde-doença e das terapias para manutenção da vida, necessárias a essa população.

### Limitações do estudo

As limitações deste estudo estão relacionadas à amostra restrita, o que pode ser justificado pelo fato de abranger apenas um centro de diálise com capacidade restrita e pequena de atendimento. Contudo, essa característica é comum aos serviços de diálise pediátrica, visto que há a ocorrência de outro método de tratamento, a DP, e essa população é flutuante, por ser prioridade para transplante. Essas limitações também são observadas em múltiplos estudos internacionais, como os referenciados, acerca dessa população e temática. Além disso, o recorte sociodemográfico é específico e regionalizado de forma que não representa as múltiplas realidades brasileiras.

### Contribuições para as áreas da enfermagem, saúde ou políticas públicas

O presente estudo apresenta uma vasta riqueza de dados referentes a questões sociodemográficas e epidemiológicas da população estudada. Além disso, faz parte de macroestudo que levanta esses e outros dados na população infantojuvenil que foi submetida à TRS durante a pandemia. Tamanha quantidade e riqueza de informação pode resultar em diversos produtos e contribuir significativamente com a produção científica em saúde nesse assunto, específico e relevante, além de fomentar mais estudos e guiar a prática assistencial.

## CONCLUSÕES

Foi identificada a soroprevalência de COVID-19 nas crianças e adolescentes em HD e em seus acompanhantes/cuidadores familiares. Destacam-se como uma importante característica identificada entre os indivíduos sororreagentes os quadros assintomáticos da doença. As variáveis sociodemográficas estudadas demonstraram a vulnerabilidade dessa população, que somada às iniquidades de acesso ao tratamento efetivo da doença renal, comprometem a qualidade de vida da criança e da família, inclusive sua sobrevida.

Guiados pelos ODS, faz-se necessária a existência de políticas públicas robustas, nacionais e internacionais, incentivadas pelos órgãos da ONU. Essas ações devem ser multissetoriais e estratégicas, atuando desde a prevenção das doenças que afetam os rins até as terapias renais substitutivas, para erradicar as iniquidades sociais e de saúde nessa população, garantindo o acesso e qualidade de assistência.

Por fim, são necessários mais estudos acerca da dinâmica viral do SARS-CoV-2 em crianças em HD para elucidar as variáveis clínicas e epidemiológicas envolvidas, além de estudos com maior abrangência de participantes, centros de diálise e regiões para demonstrar com fidedignidade a realidade da HD pediátrica no Brasil e no mundo.

## Data Availability

Os dados de pesquisa só estão disponíveis mediante solicitação.
